# Distinct Yellowfin Tuna (*Thunnus albacares*) Stocks Detected in Western and Central Pacific Ocean (WCPO) Using DNA Microsatellites

**DOI:** 10.1371/journal.pone.0138292

**Published:** 2015-09-22

**Authors:** Roselyn D. Aguila, Sweedy Kay L. Perez, Billy Joel N. Catacutan, Grace V. Lopez, Noel C. Barut, Mudjekeewis D. Santos

**Affiliations:** 1 Genetic Fingerprinting Laboratory, Research and Ecological Assessment Division, National Fisheries Research and Development Institute, Quezon City, Metro Manila, Philippines; 2 Examination Division, Laboratory Service, Philippine Drug Enforcement Agency, Quezon City, Metro Manila, Philippines; 3 Vertebrates Section, Research and Ecological Assessment Division, National Fisheries Research and Development Institute, Quezon City, Metro Manila, Philippines; 4 Office of the Interim Deputy Executive Director, National Fisheries Research and Development Institute, Quezon City, Metro Manila, Philippines; Biodiversity Insitute of Ontario - University of Guelph, CANADA

## Abstract

The yellowfin tuna, *Thunnus albacares* (Bonnaterre, 1788), covers majority of the Philippines’ tuna catch, one of the major fisheries commodities in the country. Due to its high economic importance sustainable management of these tunas has become an imperative measure to prevent stock depletion. Currently, the Philippine yellowfin tuna is believed to be part of a single stock of the greater WCPO though some reports suggest otherwise. This study therefore aims to establish the genetic stock structure of the said species in the Philippines as compared to Bismarck Sea, Papua New Guinea using nine (9) DNA microsatellite markers.

DNA microsatellite data revealed significant genetic differentiation between the Philippine and Bismarck Sea, Papua New Guinea yellowfin tuna samples. (FST = 0.034, P = 0.016), which is further supported by multilocus distance matrix testing (PCoA) and model-based clustering (STRUCTURE 2.2).With these findings, this study posits that the yellowfin tuna population in the Philippines is a separate stock from the Bismarck Sea population. These findings add evidence to the alternative hypothesis of having at least 2 subpopulations of yellowfin tuna in the WCPO and calls for additional scientific studies using other parameters to investigate this. Accurate population information is necessary in formulating a more appropriate management strategy for the sustainability of the yellowfin tuna not only in the Philippines but also in the WCPO.

## Introduction

Globally, tuna production has constantly been an important source of annual total marine production for coastal countries. In the Western and Central Pacific Ocean (WCPO), tuna species, primarily skipjack, yellowfin and bigeye tunas, have been the leading source of fishery catch and production. These three species alone contributed an approximate 2.2 million mt in the region’s fishery catch in 2011, representing 79% of the total Pacific Ocean catch [1]. In the Philippines, it has contributed 30% of the total annual marine production and 42% export share amounting to US$ 10 million [2], making it one of the country’s major fisheries commodities [3].

Sustaining tuna resources in the WCPO and in the Philippines is not only important economically but more so to the ecosystem. The Coral Triangle, a region considered to be the global center of marine biodiversity and one of the world’s top priorities for marine conservation, spanning eastern Indonesia, Malaysia, Papua New Guinea, Timor-Leste and the Solomon Islands, and the Philippines, has become a priority for monitoring and conservation, especially the marine environment [4]. Conserving this region requires that the species in it are adequately sustained to prevent imbalances that could result to stock depletion, which could have devastating effects on both biodiversity and fisheries. Thus, studies that could further identify both population connectivity and structuring are useful in improving management and conserving the region’s marine resources [5].

Though yellowfin tuna is not, as of yet, in an overfished state, it has been hugely exploited across the western equatorial Pacific thus a limit on its catch at current levels have been recommended [6,7]. Any meaningful tuna management in the WCPO requires that the tuna population stock(s) in the area be fully identified and described. Identification of existing population structures and boundaries delineated by agreeing phylogeographic distribution patterns can be used in establishing fisheries management units as well as plans for marine protected areas [5]. Yellowfin tuna (YFT), *Thunnus albacares* (Bonnaterre, 1788), is widely believed to be panmictic within and between oceans. YFT population between the Atlantic and the Pacific Oceans show low levels of genetic differentiation indicating a very slow genetic drift due to the species’ large population size [8]. Similarly, YFTs in the Western Pacific and in Western Indian Oceans showed no genetic differentiation based on non-significant pairwise FST values revealing an extensive gene flow between these ocean basins [9]. Because YFTs are oceanic and are therefore highly migratory, they are believed to be a single stock in the western and central Pacific region [9,10].

In contrast, other reports suggest that there are different YFT stocks within the Pacific Ocean. For example, in the Eastern Pacific region, the stock structure of the YFT has exhibited limited mixing between the northern and southern regions using tagging and nitrogen isotope analysis [11]. In the Western Pacific Ocean, the YFT stock has been found to have very limited heterogeneity using microsatellite markers, similar with the earlier findings using allozyme and mitochondrial DNA markers [10]. Moreover, YFT catch data as early as the 1990’s in the WCPO showed a slower growth rate along the Philippine and Indonesian waters indicating a probable population structuring [6].

Here, we compared the population of the YFT caught in the Philippine waters to the YFT population caught in Bismarck Sea, Papua New Guinea using nine (9) DNA microsatellite loci as genetic markers. YFTs in Bismarck Sea showed significant genetic heterogeneity as compared to the Philippine YFTs suggesting a separation in stocks of the two areas and the existence of at least two stocks of YFT in the WCPO.

## Materials and Methods

### Tissue Sampling, DNA Extraction, and Amplification

**Fig 1:**
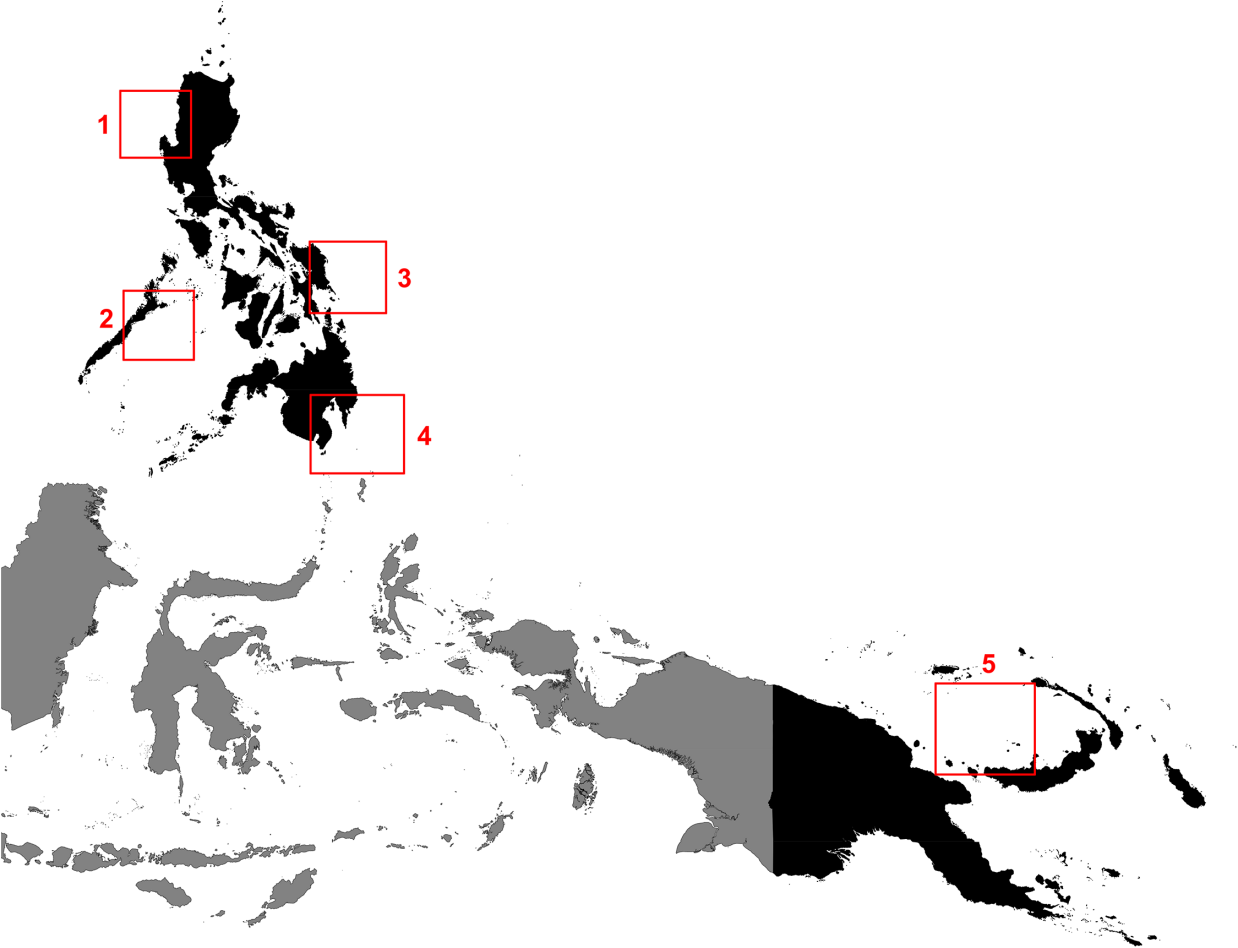
Map of Western and Central Pacific Ocean (WCPO) showing yellowfin tuna (*Thunnus albacares*) collection sites. 1 –Zambales; 2 –Palawan; 3 –Eastern Samar; 4 –General Santos; 5 Bismarck Sea, Papua New Guinea. Quantum GIS package [12] was used in the map layout of WCPO**.**

Tissue samples were extracted from 310 YFT individuals collected in the course of two years from May 2010 to May 2012 from four tuna landing sites selected across the Philippine shores and a site in the WCPO outside the Philippines (Fig 1) [12].The sample collection sites were municipal fish landing sites and nearby fish markets located in Subic, Zambales for West Philippine Sea, Puerto Princesa, Palawan for Sulu Sea, Eastern Samar for East Philippine Sea and General Santos for Celebes Sea. Representative YFT samples were collected in the Bismarck Sea, Papua New Guinea (4°17’60” S; 149°18’58” E). The Philippine samples were personally collected by the authors. The Bismarck Sea tuna samples were collected by Filipino fishing boat captains from Frabelle Fishing Corporation trained in muscle tissue collection and storage immediately after the tuna catch. No specific permits were required during sample collection since the samples are neither endangered nor protected species and the samples were collected from fishing boats and fish markets. Initial identification of the samples was based on the handbook for identifying yellowfin and bigeye tunas in fresh condition [13].

Muscle tissues were extracted from the left posterior part of the fresh or frozen YFT samples of various sizes ranging from 15 to 89 cm in fork length (S1 Table). The muscle extracts were preserved in absolute ethanol and stored at -20°C. DNA was extracted using the CTAB extraction protocol with modifications [14,15]. Nine microsatellite loci (*Obe231*, *Obe294*, *Obe652*, *Obe467*, *Obe157*, *Obe674*, *Obe527*, *Obe237*, *Obe218*, and *Obe236*) isolated from bigeye tuna were cross-amplified using the prescribed protocol [16]. Primer pairs used for PCR are listed in Table 1. Each forward primer was labeled with either 6-FAM or HEX fluorescent dye at the 5’-end. The PCR cocktail mix consisted of 0.13mM dNTPs (KAPA), 0.67μM forward and reverse primers (1^st^ BASE), 0.08U standard *Taq* polymerase (KAPA), and 1μl DNA template. The mix was aliquoted to a 12-μl reaction and was run using the following PCR parameters: initial denaturation at 95°C for 2min; 30 and 35 cycles of amplification with denaturation at 94°C for 30s, annealing at 58°C for 30s, and extension at 72°C for 30s; and a final extension at 72°C for 2min. Resulting PCR products were confirmed by running them in gel electrophoresis using 3% agarose gel. Fragment length analysis of the samples was outsourced to Macrogen Inc., Korea using the 3730XL DNA analyzer (Applied Biosystems, USA) and Big Dye Terminator v3.1 Cycle Sequencing Kit (Applied Biosystems, USA) with size standards 400-HD and 500-LIZ.

**Table 1:** Forward and reverse 5'-3' primer sequences used in PCR amplification of ten DNA microsatellite markers [15].

Locus	Forward Primer Sequence (5'-3')	Reverse Primer Sequence (5'-3')
Obe 157	TTCTCTGGCTGAATGCTGTC	TTGTCAACGAAGGTGAACACA
Obe 218	GGCGTAGGTCCACTCACATT	TGCCTGCTGTTTTACCAAGA
Obe 231	GTGGCCCTCTGTGAAACTGT	ATCATCATCGCTGCCTCTCT
Obe 236	CCATGTTTTCACACAATTTTCAA	TGACCTGCTGACACAGGAAG
Obe 237	TCTAAGGGAACCAGCGAGAA	TAGCATCAACAGAGGCCAAA
Obe 294	CCAGGGCTCCTGATTCTGAT	TCACATTCCTTGACCCATTT
Obe 457	GCAGCAACACAGAGACAGGA	GGATCCCCACGAGGACTACT
Obe 527	CCTTCAGGACCTGTCAGGAG	CTTTCTGTCTGCTCCGTTCC
Obe 652	TGAGTGGCAGGCAGTAAGTG	CAAGCTCGACGCAATTACAA
Obe 674	TATCATGGGTCGGGTCCTAA	GGGGCTCTCTCAATCCTACC

### Genetic Analysis

Prior to statistical analyses, the samples were identified as YFTs by running phylogenetic trees (Neighbor-Joining and maximum likelihood using the Tamura-Nei model with gamma value = 0.576) in MEGA5 [17] using partial fragment sequences of the mtDNA D-loop control region against the YFT sequences (GenBank accession numbers JN988636.1 –JN988641.1) of Pedrosa-Gerasmio et al. [18]. Representative bigeye sequences (GenBank accession numbers JN988645.1 –JN988649.1) from the same study were included as outgroup (S1 and S2 Figs). Calling alleles obtained from fragment analysis was done using Peak Scanner software v1.0 from Applied Biosystems by Life Technologies [19] (S2 Table). Allele size frequencies were computed using Excel Microsatellite Toolkit v.31 [20]. Genetic variation in microsatellite loci in the five populations was analyzed by determining the number of alleles per locus (a), allelic richness (Rs), observed heterozygosity (Ho), and expected heterozygosity (He) for each locus from each site using GENEPOP v4 [21]. The same program was also used to check for deviations from the Hardy-Weinberg equilibrium (HWE) and linkage disequilibrium of each locus within each site (exact tests; [21]). The estimates of Wright’s FST to evaluate significant genetic variation between the populations being observed were calculated using ARLEQUIN v3.5.1.2 [22–25]. The significance of all statistical analyses was assessed using an adjusted alpha by the sequential Bonferroni procedure [26]. Principal Coordinates Analysis of the multilocus data among the five sampling locations was calculated and graphed using GenAlex 6.5 [27,28]. A model-based clustering method for inferring population structure of yellowfin tuna was implemented in STRUCTURE 2.2 [29]. The samples were tested with 10 values of K (K = 1 to K = 10) each for ten iterations using the Admixture model with inferred alpha and correlated allele frequencies at set lambda = 1. The most suitable K was inferred using the Evanno method [30] employed in the program Structure Harvester [31].

## Results

### DNA Microsatellite Variation

Yellowfin tuna samples of sizes ranging from 15 to 89 cm in fork length (S1 Table) were collected from public markets and municipal landing sites along the four major seaboards of the Philippines and from Bismarck Sea, Papua New Guinea with the cooperation of Frabelle Fishing Corporation, a Philippine fleet fishing in the high seas. Ten microsatellite loci were analyzed for significant variation among the yellowfin tuna samples. These loci were tested for deviation against the Hardy-Weinberg Equilibrium (HWE) to avoid the use of non-neutral locus. One of these ten loci, *Obe652*, was found to significantly deviate from HWE, thus was not used in the rest of the analysis. The allelic richness observed from each sample site using these nine loci ranges from two to 13 alleles. Observed heterozygosities range from 0.182 to 0.867 as compared to the sample population’s expected heterozygosities ranging from 0.355 to 0.861. Basic descriptive statistics of each locus in each sample site are shown in Table 2. Allele frequencies of each locus globally and in each sample site are shown in Tables A-J of S1 File.

**Table 2:** Descriptive statistics of nine microsatellites in *Thunnus albacares*.

	Locus										Average across loci
Sample Location	Obe 218	Obe 236	Obe 231	Obe 294	Obe 652	Obe 467	Obe 157	Obe 674	Obe 527	Obe 237	
**General Santos**											
*N*	50	50	46	45	45	47	48	49	49	49	
*A*	6	13	9	13	7	4	8	6	3	7	7.6
*Rs*	5.976	12.491	8.819	12.659	6.993	3.990	7.625	5.835	2.857	6.712	7.396
*He*	0.705	0.826	0.806	0.841	0.675	0.617	0.638	0.507	0.482	0.566	0.666
*Ho*	0.620	0.800	0.717	0.867	0.356	0.574	0.604	0.551	0.408	0.469	0.597
*HW*	0.556	0.291	0.071	0.472	0.000	0.021	0.329	0.585	0.225	0.150	
**Eastern Samar**											
*N*	49	50	46	44	44	47	47	50	50	50	
*A*	7	12	9	12	6	4	5	5	3	8	7.1
*Rs*	6.857	11.628	8.739	11.862	5.998	3.894	4.990	4.792	2.840	7.628	6.923
*He*	0.721	0.853	0.796	0.857	0.762	0.624	0.533	0.505	0.473	0.555	0.668
*Ho*	0.653	0.780	0.717	0.818	0.182	0.681	0.511	0.560	0.400	0.420	0.572
*HW*	0.270	0.321	0.692	0.114	0.000	0.765	0.313	0.756	0.484	0.091	
**Palawan**											
*N*	42	42	42	42	42	42	42	43	43	43	
*A*	7	10	8	12	5	3	7	5	4	8	6.9
*Rs*	7.000	10.000	8.000	12.000	5.000	3.000	7.000	4.976	3.953	7.907	6.884
*He*	0.643	0.838	0.754	0.846	0.717	0.547	0.650	0.355	0.467	0.518	0.634
*Ho*	0.667	0.762	0.690	0.857	0.214	0.500	0.714	0.372	0.465	0.419	0.566
*HW*	0.492	0.519	0.230	0.834	0.000	0.087	0.156	0.035	0.618	0.101	
**Zambales**											
*N*	50	48	49	49	49	44	44	50	50	50	
*A*	7	10	10	10	6	4	7	5	2	9	7
*Rs*	6.816	9.873	9.671	9.711	5.981	3.998	6.953	4.812	2.000	8.607	6.842
*He*	0.732	0.861	0.773	0.836	0.708	0.617	0.655	0.426	0.447	0.576	0.663
*Ho*	0.680	0.708	0.796	0.776	0.327	0.682	0.614	0.400	0.460	0.560	0.600
*HW*	0.746	0.029	0.771	0.545	0.000	0.891	0.268	0.253	1.000	0.280	
**Bismarck Sea, PNG**											
*N*	44	43	45	45	44	44	44	39	39	46	
*A*	12	19	13	13	6	8	9	3	2	6	9.1
*Rs*	11.747	18.510	12.583	12.580	5.998	7.875	8.659	3.000	2.000	5.994	8.895
*He*	0.838	0.921	0.899	0.870	0.733	0.819	0.705	0.429	0.441	0.496	0.715
*Ho*	0.818	0.884	0.889	0.800	0.545	0.863	0.545	0.487	0.385	0.522	0.674
*HW*	0.382	0.307	0.463	0.000	0.009	0.946	0.068	0.511	0.471	0.356	

*n–*sample size; *a*–number of alleles per locus; *Rs–*allelic size range; *He–*expected heterozygosity; *Ho*–observed heterozygosity; *HW–*deviation from Hardy-Weinberg equilibrium

Hierarchical variations using distance method based on the number of different alleles for the whole population are presented in Table 3. No significant genetic differentiation was observed in all hierarchies. The single stock assumption of the Philippine sites was inferred from this data albeit inconclusive since the samples were mostly juvenile collected from markets and municipal landing sites and do not account for adults that could be migrating around the Philippine waters. The YFT samples were then treated *a priori* as two groups, the pooled Philippine sites and the Bismarck Sea, Papua New Guinea site. Significant genetic differentiation was observed between these two groups with FST = 0.034 (*P* = 0.016). This finding between the pooled Philippine samples and the Bismarck Sea samples is considered moderate variation based on Wright’s qualitative guideline [32].

**Table 3:** Analysis of Molecular Variance (AMOVA) of genetic variation of yellowfin tuna from five locations.

Source of variation	Sum of squares	Variance components	Percentage variation
**Among groups**	102.58	0.704	16.44
**Among populations within groups**	13.48	0.006	0.14
**Among individuals within populations**	881.31	0.362	8.46
**Within individuals**	736.00	3.210	74.95

Genetic differentiation estimates were also determined between pairs of sites using distance method based on the number of different alleles. Samples from the Philippine sites were compared to those from Bismarck Sea, Papua New Guinea to confirm whether these sites exhibit structuring, in an attempt to support the variation observed between the two groups.

On the other hand, Significant FSTs were observed in Bismarck Sea, Papua New Guinea when paired to the four Philippine sites with FST ranging from 0.2233 to 0.2582, exhibiting moderate to great variation (P = 0.00000; Table 4). Among the significant pairwise estimates, the Zambales-Bismarck Sea pair presented the highest degree of differentiation at FST = 0.2382 (*P =* 0.00000).

**Table 4:** Population pairwise FSTs (lower diagonal) and P-values (upper diagonal) of *T*. *albacares* using between the five locations.

Location	General Santos	Samar	Palawan	Zambales	Bismarck Sea, PNG
**General Santos**	–	0.8018	0.3243	0.4865	0.0000**
**Samar**	-0.0024	–	0.1441	0.1892	0.0000**
**Palawan**	-0.0006	0.0039	–	0.1982	0.0000**
**Zambales**	0.0003	0.0071	0.0035	–	0.0000**
**Bismarck Sea, PNG**	0.2233	0.2274	0.2582	0.2382	–

Distance method based on number of different alleles of nine microsatellite loci

**Significant at α = 0.05

### Genetic Structuring

**Fig 2:**
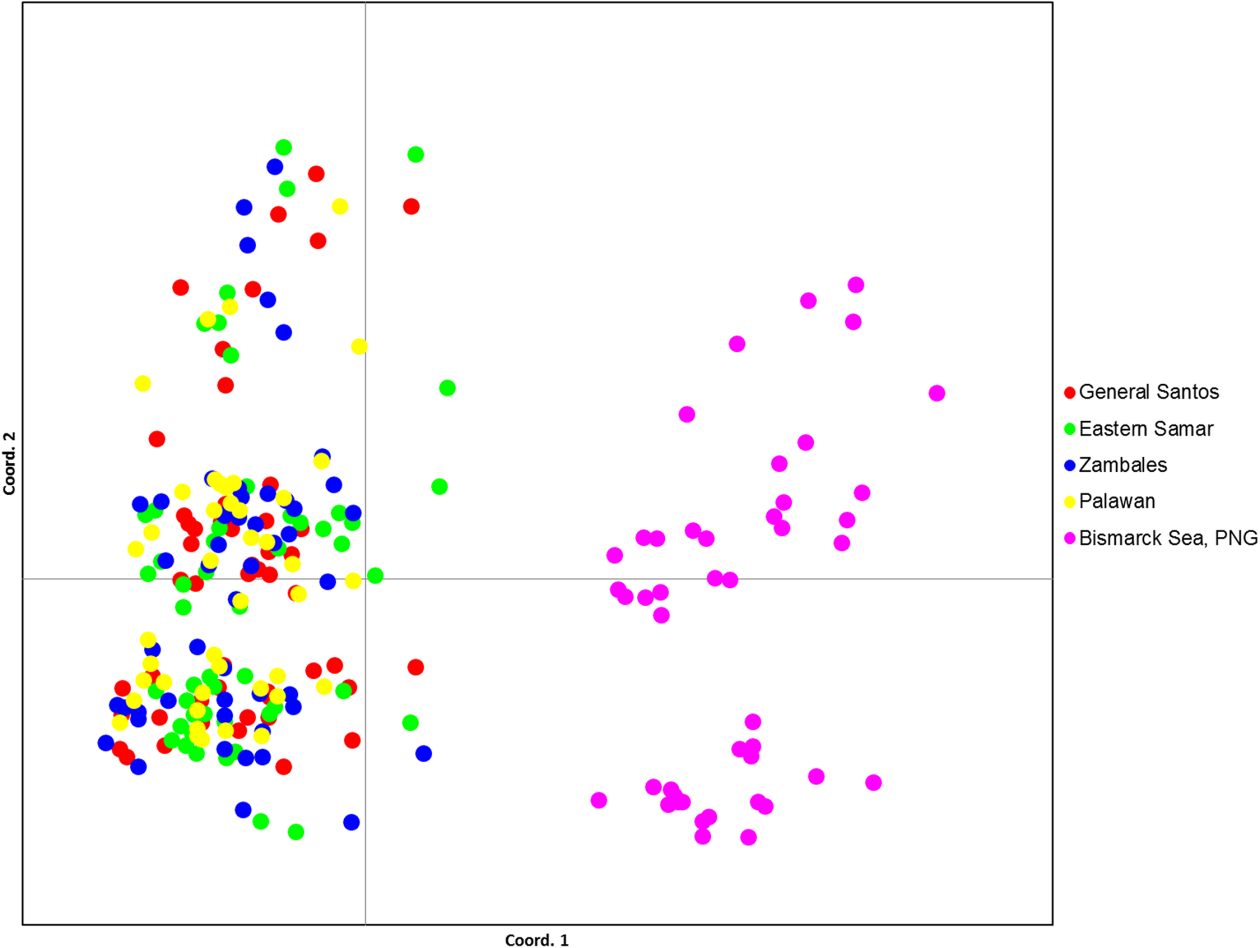
Principal Coordinates Analysis of *T*. *albacares* exhibiting two separate clusters based on district matrix using nine DNA microsatellite loci. Red–General Santos; Green–Eastern Samar; Blue–Zambales; Yellow–Palawan; Purple–Bismarck Sea, Papua New Guinea.

DNA microsatellite variation is supported by the separation of the samples into two distinct groups as observed in the Principal Coordinates Analysis (PCoA) using a multilocus distance matrix as represented in Fig 2. Each colored dot represents a yellowfin individual collected in a corresponding sampling site as indicated by its corresponding color. The YFT samples collected from Bismarck Sea, Papua New Guinea (purple dots) formed a distinct group on the right axis of the plot, separate from samples collected in the Philippine sites. This suggests a distinct clustering between the YFT samples caught in the Philippine sites and Bismarck Sea, Papua New Guinea.

**Fig 3:**
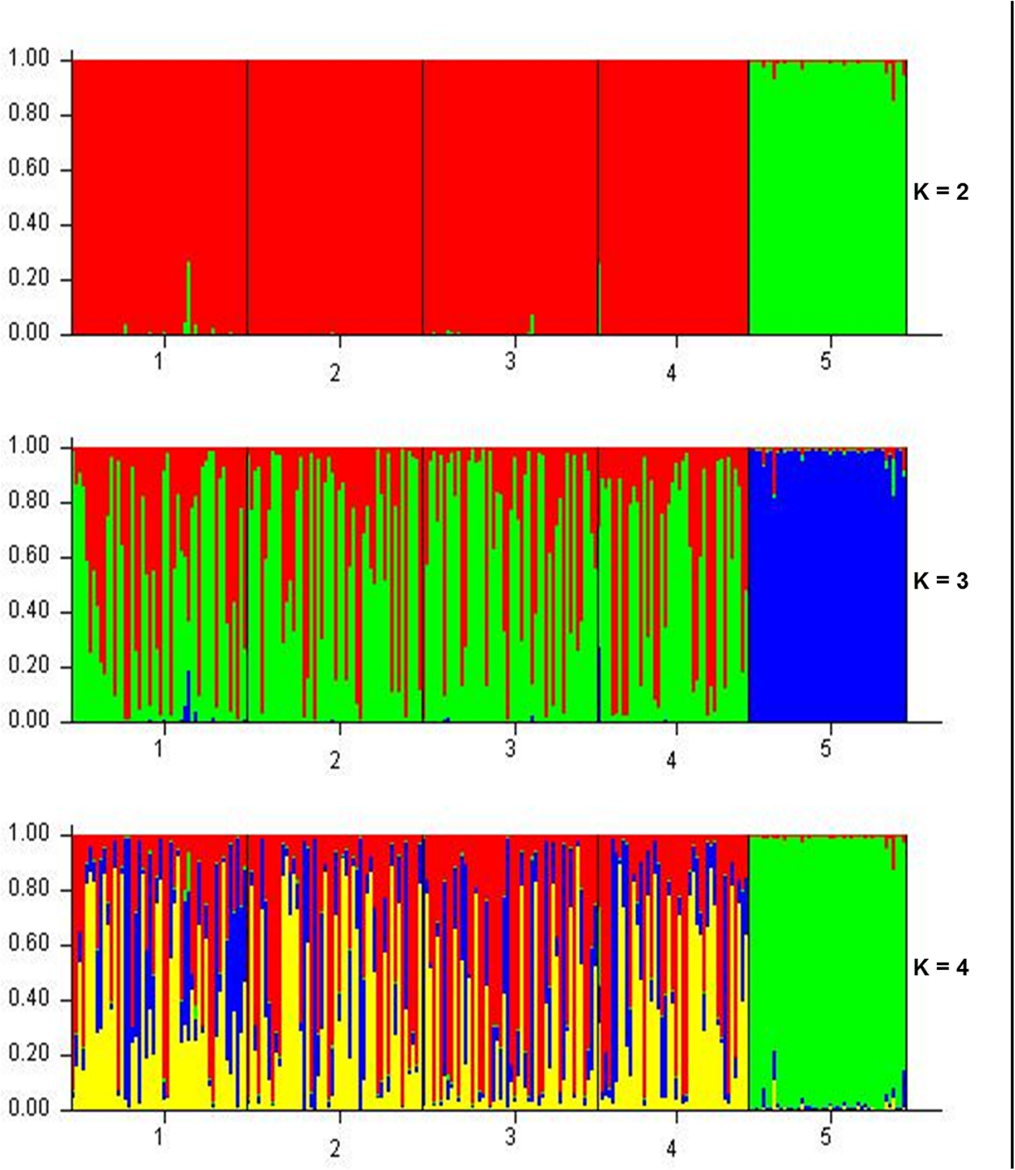
Bar plots of different assumptions of clusters, K in *T*. *albacares* based on multilocus data. Plots for values of K = 1 to K = 10 were constructed in STRUCTURE 2.2, with 10 replicate runs for each K value. Plots for the most significant K values, K = 2 to K = 4, are shown.

**Fig 4:**
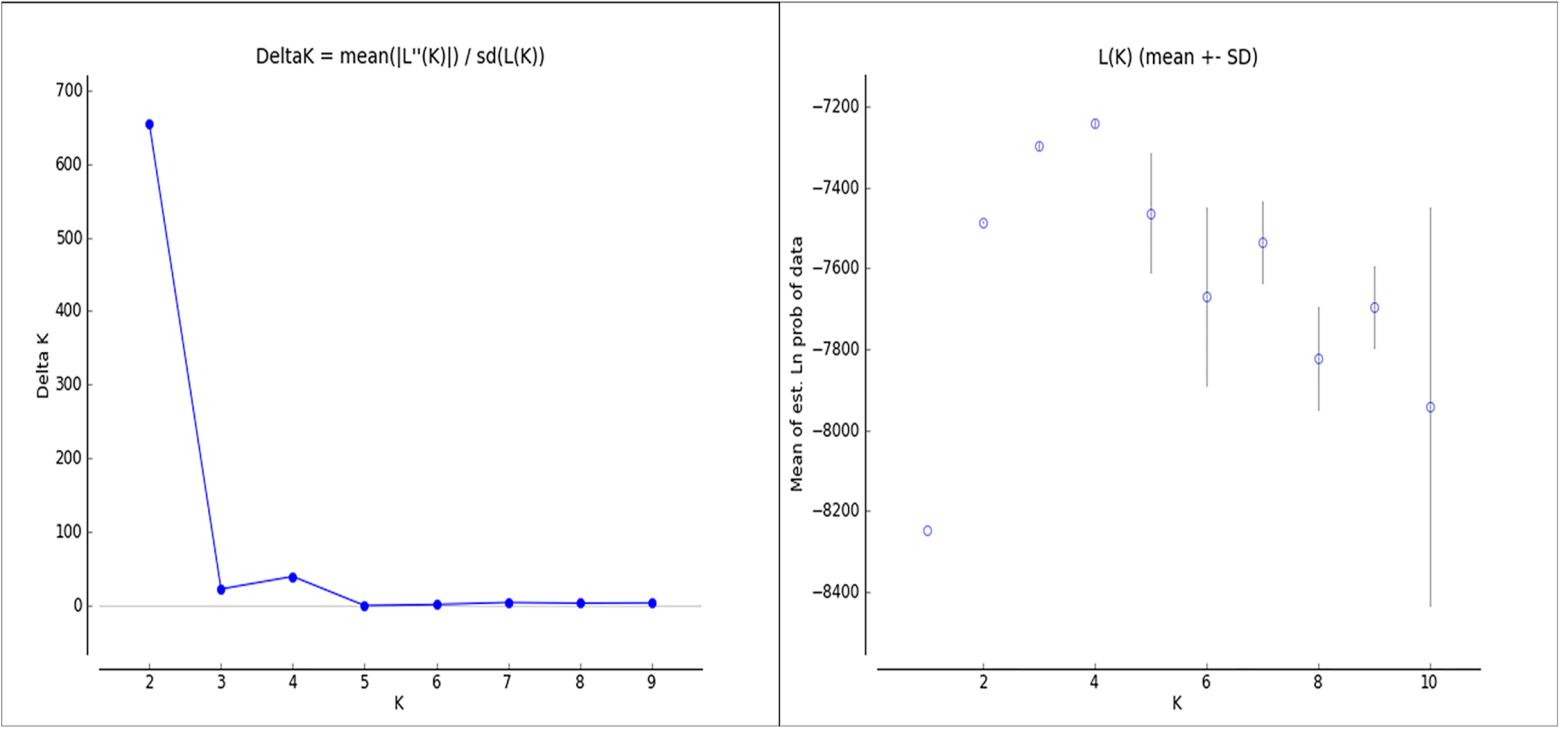
Delta K and the mean of estimate natural log probability of STRUCTURE runs of *T*. *albacares* samples using values of K = 1 to K = 10.

Further support on the distinct clustering of YFT was obtained upon using the model-based clustering method, STRUCTURE, on our multilocus genotype data. Ten values of K (S3 Fig), with 10 iterations for each K value, were tested as shown in Fig 3. The most suitable value of K was assessed using Delta K, a statistic that is based on the rate of change in the log probability of data in a series of K values [30]. The most suitable value of K is K = 2 (Fig 4) and was thus used in interpreting the clustering result of the analysis. The numbers in the bar plot (Fig 3, K = 2) corresponds to the sampling site from which the individuals were collected. All four plots representing Philippine sites, 1–4, were marked red, indicating one stock. Meanwhile, the plot representing the Bismarck Sea samples, site 5, was marked green. This indicates difference in structure compared to the YFT samples from the Philippine sites which were marked red.

## Discussion

Fisheries conservational management has been a main concern for coastal countries like the Philippines in the past few decades. Strategies have been implemented over the years in sustaining marine stocks especially the commercially important organisms. Technologies have also been enhanced in an effort to aid in creating and improving existing management strategies especially in the wider marine systems. Among these technologies, the advent of the more stable genetic markers in inferring marine system connectivities has been one of the greatest breakthroughs in population studies. Though not an absolute deciding factor in delineating subpopulations among organisms, identifying an organism’s genetic stock structure has become a key in determining other equally important factors like gene flow, migration and dispersal with which a stock may be concretely determined. Specifically, these genetic markers can provide hints on the connectivity of stocks of marine organisms that can be further used in designing and redesigning sustainable management strategies [33]. Studies have been conducted over the years to look into the stock structure of tunas, one of the most important marine stocks, in ocean basins worldwide, employing different methods including allozymes [34,35], restriction fragment length polymorphism (RFLP) markers [8,36,37], mitochondrial DNA markers [9,38–40] and microsatellite markers [10,41].

Analyses of molecular variance using suitable genetic distance methods were conducted to confirm whether the Philippine stock is separate from the widely believed single WCPO stock. There was no significant differentiation observed among the YFT samples from the four Philippine sites based on DNA microsatellite data albeit inconclusive due to sampling limitations. Philippine was then compared to Bismarck Sea YFTs which represents part of the WCPO. Moderate genetic differentiation was observed between the two areas based on both genetic distances and pairwise differences. Similarly, comparison between the Bismarck Sea YFTs and each group of samples from different Philippine sites yielded significant variation based on both genetic distances and pairwise differences. The Zambales-Bismarck Sea pair, which has the highest degree of differentiation observed, was the most significant among the pairwise estimates due perhaps to the Zambales fishing site which is within the West Philippine Sea and is geographically separated from the Pacific Ocean by the Philippine archipelago. These evidences further strengthen the hypothesis that the Philippines might have a single stock that is separate from the greater Western and Central Pacific stock, contrary to the current assumption that the region only has a single stock of yellowfin tuna. To further support this assumption, clustering into two YFT stocks were observed in both PCoA using distance matrix and model-based clustering method, STRUCTURE. Both analyses clearly delineated the two distinct groups observed in both tests for genetic distances and pairwise differences.

Having a Philippine stock of yellowfin tuna separate from the Western and Central Pacific is possible because of the presence of biogeographic barriers such as eddies and upwellings as well as strong ocean currents like the North Equatorial Current on Philippine borders. Jackson et al. [42], suggested that the Mindanao eddies could act as barriers to larval dispersal that causes to maintain genetic divergence among pelagic fish stocks in the area. Not surprisingly therefore, not much movement were observed in tagged Philippine tunas going out to adjacent areas [43]. This restriction was attributed to the Philippine bathymetry, preventing the tunas to cross to nearby areas. Moreover, yellowfin tuna stock in the then WCPO region 3 in which the Philippines is included, exhibited biological differences, i.e. having slower growth rates, as compared to the tuna stock of the rest of the WCPO [6]. Such variability in growth between stocks may be indicative of their difference in addition to genetic variation, as have been observed in brown trout [44].

A recent study on yellowfin tuna also reported a possible admixture of Taiwan stock to the Philippine Sea stock, although only one geographic location was sampled [45]. Population structuring and concordant barriers in larval dispersal in neritic tunas were also observed within the Indonesian waters as caused by Pleistocene vicariance [42]. Other studies in another tuna species, the skipjack tuna (*Katsuwonus pelamis*), also support this divergent tuna stocks scenario, as they revealed a possible stock delineation in the Western and Central Pacific Ocean using serum esterase & transferrin system allozymes [34,35].

## Conclusion

The analysis of these YFT samples using DNA microsatellite markers exhibited moderate variation between the pooled Philippine samples and the Bismarck Sea samples. This strongly suggests the existence of a distinct YFT stock in the Philippines different from the YFT stock found in the Bismarck Sea, Papua New Guinea.

To further support the findings of this study, it is recommended that further studies should include additional sampling sites from both areas of Papua New Guinea and the Philippines and eventually the rest of the Western and Central Pacific region. Additionally, larval sampling may be added as the source of genetic material. Other parameters to test the stock structure of the yellowfin tuna in the WCPO could be included such as the use of length frequencies, reproductive biology, otolith, parasites, tagging etc. The accuracy of such population structure approach will greatly help in assessing stock status and determining the most suitable management strategy for the region’s yellowfin tuna.

## Supporting Information

S1 Fig Neighbor-Joining tree of *T*. *albacares* partial D-loop fragment sequences using Tamura-Nei model (gamma value = 0.576) with 500 bootstrap replications to confirm correct species identification. A subsample (n = 73) was analyzed with the *T*. *albacares* partial D-loop fragment sequences of Pedrosa-Gerasmio et al. (2012) to confirm the correct species identification of the sampled individuals. Representative bigeye tuna sequences from the same study were used as outgroup.
(TIF)

S2 Fig Maximum likelihood tree of *T*. *albacares* partial D-loop fragment sequences using Tamura-Nei model (gamma value = 0.576) with 500 bootstrap replications to confirm correct species identification. A subsample (n = 73) was analyzed with the *T*. *albacares* partial D-loop fragment sequences of Pedrosa-Gerasmio et al. (2012) to confirm the correct species identification of the sampled individuals. Representative bigeye tuna sequences from the same study were used as outgroup.
(TIF)

S3 Fig Bar plots of different assumptions of clusters, K in *T*. *albacares* based on multilocus data. Plots for values of K = 1 to K = 10 were constructed in STRUCTURE 2.2, with 10 replicate runs for each K value.
(TIF)

S1 File Allele size frequency tables for all populations of *T*. *albacares* by locus. Allele size frequencies of Locus Obe218 for all populations of *T*. *albacares* **(Table A)**. Allele size frequencies of Locus Obe236 for all populations of *T*. *albacares* **(Table B)**. Allele size frequencies of Locus Obe231 for all populations of *T*. *albacares* **(Table C)**. Allele size frequencies of Locus Obe294 for all populations of *T*. *albacares* **(Table D)**. Allele size frequencies of Locus Obe652 for all populations of *T*. *albacares* **(Table E)**. Allele size frequencies of Locus Obe467 for all populations of *T*. *albacares* **(Table F)**. Allele size frequencies of Locus Obe157 for all populations of *T*. *albacares* **(Table G)**. Allele size frequencies of Locus Obe674 for all populations of *T*. *albacares* **(Table H)**. Allele size frequencies of Locus Obe527 for all populations of *T*. *albacares* **(Table I)**. Allele size frequencies of Locus Obe237 for all populations of *T*. *albacares* **(Table J)**.
(XLSX)

S1 Table Sizes of *T*. *albacares* individuals. Fork length of each sample was measured in cm (±1.0).
(XLSX)

S2 Table Allele sizes of *T*. *albacares* individuals per locus. Allele sizing was done using Peak Scanner software v1.0. Samples were coded according to their sampling location sites: YFG—General Santos; YFS—Samar; YFZ—Zambales; YFP—Palawan; YFBS—Bismarck Sea, Papua New Guinea.
(XLSX)
