# Correction: Distinct Yellowfin Tuna (*Thunnus albacares*) Stocks Detected in Western and Central Pacific Ocean (WCPO) Using DNA Microsatellites

**DOI:** 10.1371/journal.pone.0144568

**Published:** 2015-12-03

**Authors:** Roselyn D. Aguila, Sweedy Kay L. Perez, Billy Joel N. Catacutan, Grace V. Lopez, Noel C. Barut, Mudjekeewis D. Santos

There is an error in affiliation 2 for author Sweedy Kay L. Perez. Affiliation 2 should be: Genetic Fingerprinting Laboratory, National Fisheries Research and Development Institute, Quezon City, Metro Manila, Philippines.
