# Supplementary figures and images for: Distinct Yellowfin Tuna (Thunnus albacares) Stocks Detected in Western and Central Pacific Ocean (WCPO) Using DNA Microsatellites
Source: PLoS One. 2015 Sep 22;10(9):e0138292. doi: 10.1371/journal.pone.0138292 (PMC4634617; doi:10.1371/journal.pone.0138292)

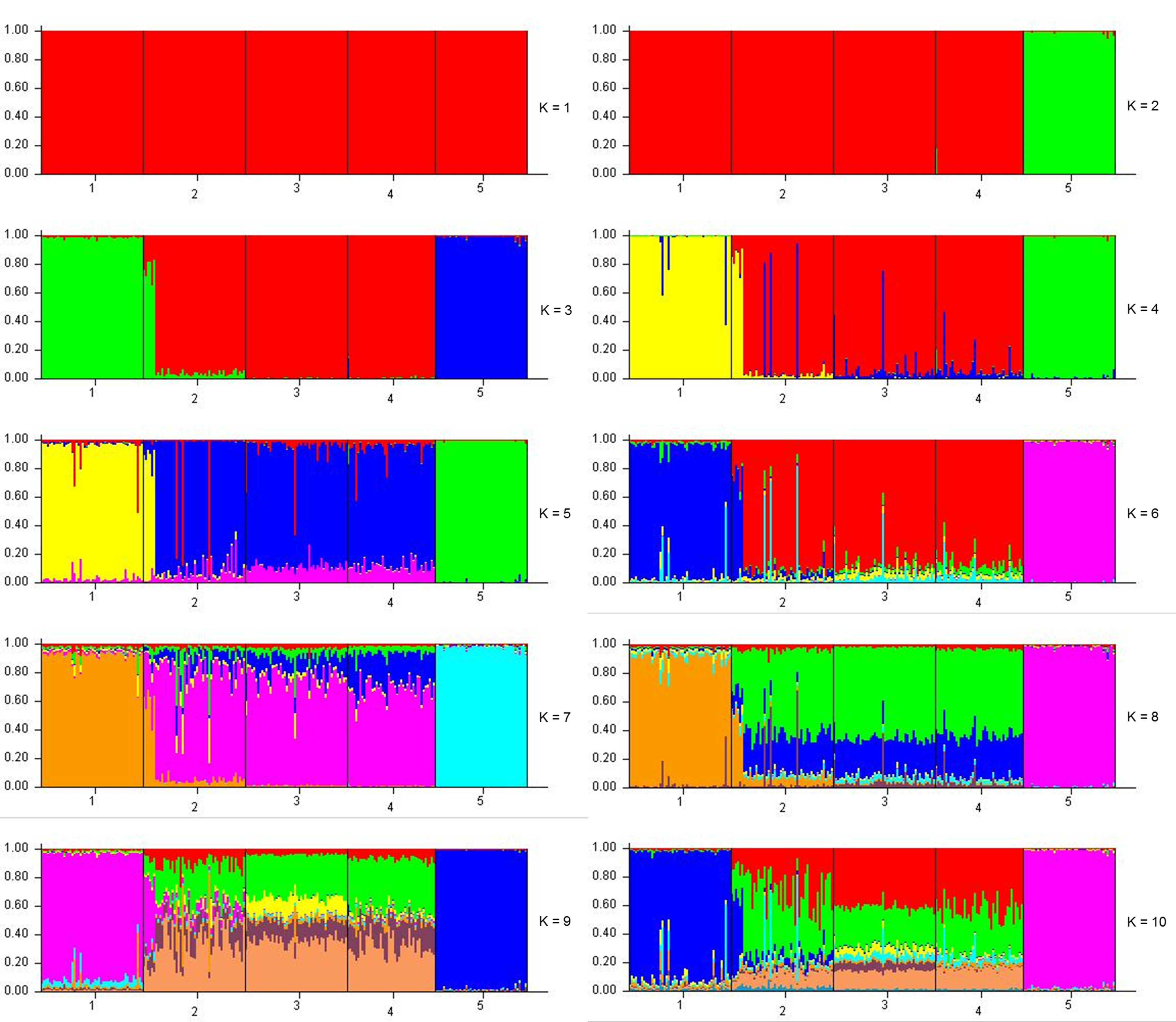

Supplement: S3 Fig — Plots for values of K = 1 to K = 10 were constructed in STRUCTURE 2.2, with 10 replicate runs for each K value. (TIF) [file pone.0138292.s003.tif]
